# Development and Characterizations of Pullulan and Maltodextrin-Based Oral Fast-Dissolving Films Employing a Box–Behnken Experimental Design

**DOI:** 10.3390/ma15103591

**Published:** 2022-05-18

**Authors:** Kiramat Ali Shah, Binbin Gao, Robia Kamal, Anam Razzaq, Sun Qi, Qiu-Ning Zhu, Song Lina, Linyu Huang, Grainne Cremin, Haroon Iqbal, Farid Menaa, Jing-Hao Cui

**Affiliations:** 1 College of Pharmaceutical Sciences, Soochow University, Suzhou 215123, China; 20207226002@stu.suda.cn (K.A.S.); robiasudozai20@gmail.com (R.K.); anamrazzaq.ajk@gmail.com (A.R.); sunqi08232022@163.com (S.Q.); 20184226013@stu.suda.edu.cn (Q.N.Z.); 20204226041@stu.suda.edu.cn (S.L.); 20205226006@stu.suda.edu.cn (L.H.); 2 Department of Molecular and Cellular Therapeutics, Royal College of Surgeons in Ireland (RCSI), D02YN77 Dublin, Ireland; binbingao20@rcsi.com (B.G.); gcremin@tcd.ie (G.C.); 3 The Cancer Hospital of the University of Chinese Academy of Sciences (Zhejiang Cancer Hospital), Institute of Basic Medicine and Cancer (IBMC), Chinese Academy of Sciences, Hangzhou 310022, China; harooniqbal415@hotmail.com or; 4 Department of Internal Medicine and Nanomedicine, California Innovations Corporation, San Diego, CA 92037, USA

**Keywords:** oral fast-dissolving film, maltodextrin, pullulan, propylene glycol, Zolmitriptan, drug delivery

## Abstract

Migraine is a neurological disorder characterized by severe headaches, visual aversions, auditory, and olfactory disorders, accompanied by nausea and vomiting. Zolmitriptan (ZMT^®^) is a potent 5HT1B/1D serotonin receptor agonist frequently used for the treatment of migraine. It has erratic absorption from the gastrointestinal tract (GIT), but its oral bioavailability is low (40–45%) due to the hepatic metabolism. This makes it an ideal candidate for oral fast dissolving formulations. Hence, the current study was undertaken to design and develop oral fast-dissolving films (OFDFs) containing ZMT for migraine treatment. The OFDFs were formulated by the solvent casting method (SCM) using Pullulan (PU) and maltodextrin (MDX) as film-forming agents and propylene glycol (PG) as a plasticizer. The strategy was designed using Box–Behnken experimental design considering the proportion of PU:MDX and percentage of PG as independent variables. The effectiveness of the OFDF’s was measured based on the following responses: drug release at five min, disintegration time (D-time), and tensile strength (TS). The influence of formulation factors, including percent elongation (%E), thickness, water content, moisture absorption, and folding endurance on ZMT-OFDFs, were also studied. The results showed a successful fabrication of stable ZMT-OFDFs, with surface uniformity and amorphous shape of ZMT in fabricated films. The optimized formulation showed a remarkable rapid dissolution, over 90% within the first 5 min, a fast D-time of 18 s, and excellent mechanical characteristics. Improved maximum plasma concentration (C max) and area under the curve (AUC 0–t) in animals (rats) treated with ZMT-OFDFs compared to those treated with an intra-gastric (i-g) suspension of ZMT were also observed. Copolymer OFDFs with ZMT is an exciting proposition with great potential for the treatment of migraine headache. This study offers a promising strategy for developing ZMT-OFDFs using SCM. ZMT-OFDFs showed remarkable rapid dissolution and fast D-time, which might endeavor ZMT-OFDFs as an auspicious alternative approach to improve patient compliance and shorten the onset time of ZMT in migraine treatment.

## 1. Introduction

Migraine is a long-lasting, agonizing and relapsing neurological disorder, affecting 10 out of every 100 individuals worldwide [1]. This impressive number enacts a substantial socioeconomic burden in terms of high medical expenses, psychosocial disability, and unemployment. Patients with migraine display an extensive array of visual, auditory, olfactory disorders, nausea and vomiting [2]. In addition, migraine is associated with neurosis, dementia, tetraplegia, and other devastating neurologic conditions. In relieving migraine, patients strive for quick relief, and typical concerns include fast absorption and rapid onset of drug action [3].

Zolmitriptan (ZMT) is a 5HT1B/1D serotonin receptor agonist of BCS class III with high water solubility and poor biofilm permeability. It is a gold standard for treating migraine and cluster headache [4]. It acts by constricting dilated blood vessels and curbing vasoactive neuropeptide release, thereby relieving migraine pain [5]. It is commercially available as fast-dissolving tablets (2.5 mg), conventional tablets (2.5 mg, 5 mg), and nasal spray (5 mg). Despite its potency, ZMT oral dosage forms have some drawbacks, such as fear of choking, large size, swallowing difficulty, low bioavailability (40%), slow onset of action (45 min), as well as other significant individual differences [6]. Moreover, symptoms, such as stomachache, nausea, and vomiting, are closely related to migraine and could affect oral medications and ultimately absorption efficiency [2]. ZMT nasal sprays are similarly problematic due to a short half-life; hence, repeated doses are needed which could lead to patient incompliance or damage to the nasal mucosa and cilia [7]. Therefore, to overcome the limitations concomitant with the available dosage form of ZMT, it is highly desirable to optimize an alternative method, such as oral fast-dissolving films (OFDFs). 

Oral fast-dissolving films (OFDFs) are relatively new dosage forms that deliver drug moiety via the oral cavity or oromucosal route. They have recently been used in geriatrics, pediatrics, and patients with either physiological or psychosomatic-induced dysphagia, to a great effect [8]. This is because OFDFs can be used without solvent intake. OFDFs attractiveness as drug delivery systems (DDS) is further emphasized by fast onset of action, high patient compliance, ease of conveyance and handling, and circumvention of the first-pass metabolism over other directly swallowed oral dosage forms [9]. Moreover, OFDFs may enhance flexibility, portability, and ease of swallowing; offering little risk of choking compared to most oral dissolving tablets (ODTs) [10,11]. However, OFDFs also have some limitations due to the disparity in formulation, which can result in poor mechanical properties, such as surface blistering, mold peeling difficulties, occasional wrinkles, or cracks. In addition, the existence of solvent residues and prolonged or altered rates of disintegration and dissolution impede the manufacturing and clinical application of OFDFs [11,12]. Thus, it is necessary to carry out systematic research to optimize the formulation of OFDFs. Numerous hydrophilic polymers are employed as film formers for OFDFs, such as polyvinyl alcohol (PVA) [13], pullulan [14], maltodextrin [15], hydroxypropyl methyl cellulose (HPMC) [16], and Kollicoat^®^ IR [11], cyclodextrins [17], carbon nanomaterials [18], mesoporous silica [19], and many others. 

Moreover, pullulan (PU) is a water-soluble straight-chain polysaccharide in which α-1,4, and α-1,6 glycosidic linkages connect the glucose units of maltotriose. Its molecular weight is roughly 200,000 Dalton with 480 maltotrioses [20]. The aptness of PU for OFDFs is being investigated because of its unique traits, such as film plasticity, viscosity, water solubility, and biodegradability. Despite its noteworthy adaptability and inimitable features, there are drawbacks to PU-based OFDFs. PU can cause films to become brittle and would require further optimization before being incorporated into their design [21]. Moreover, this PU is expensive due to its relatively limited sources. Although accomplishing consistent polymer blends with desirable properties is relatively difficult, the blending of PU with other low-cost compatible polymers offers a low-cost alternative to the development of OFDFs system with improved physicochemical properties. In the recent past, other edible polymers, such as HPMC [22], starch [23], and chitosan (CS) [24,25], have been considered as a co-polymer with PU, to achieve OFDFs with desirable physicochemical and mechanical properties. Therefore, a compatible and cost-effective maltodextrin (MDX) is proposed in this study.

MDX is known as water-soluble dextrin and can be produced from various starch forms. The properties of MDX depend on their dextrose equivalent value (DE value < 20%). With a molecular weight of around 684–6840 Daltons, it can quickly diffuse and dissolve in water, making it an ideal choice for use in OFDFs. MDX has a particular film-forming competence, but the mechanical characteristics of MDX-based OFDFs are inconsistent [26]. Therefore, improved physicochemical and mechanical properties are expected by combining PU with MDX in the film to improve the therapeutic demands of ZMT-OFDFs for migraine relief. However, the application of PU and MDX as the main film-forming components has been sparsely explored. To the best of our knowledge, there is a lack of information about the impacts of an individual polymer or polymer combination with plasticizers on the physicochemical and mechanical properties of OFDF formulations.

The study was designed to develop patient-friendly ZMT-OFDFs with the ultimate intent to improve the physicochemical and mechanical characteristics of films by integrating ZMT, MDX, and PU into a single film. The Box–Behnken design was applied to optimize OFDFs with a rapid dissolution rate, fast disintegration, and favorable mechanical properties. In addition, the optimized formulation was used for in vivo assessment, in rats, in comparison with i-g suspension.

## 2. Materials and Methods

### 2.1. Materials

ZMT was obtained from Energy Chemical Co., Ltd. (Shanghai, China). PU and MDX were purchased from Freda Biotechnology Co., Ltd. (Linshu County, Linyi, China) and COFCO biochemical energy Co., Ltd. (Longjiang County, Qigihaer, China), respectively. Propylene glycol (PG) and polyethylene glycol 400 (PEG) were obtained from Yonghua Chemical Technology Co., Ltd. (Zhitang District, Changshu, China) and Ling Feng Chemical Reagent (Shanghai, China), respectively. Trimethylamine, glycerin (GLY), anhydrous sodium hydrogen phosphate, and potassium dihydrogen phosphate were received from Sinopharm Chemical Reagent Co., Ltd. (Shanghai, China). HPLC grade acetonitrile and methanol were bought from Fisher Scientific Worldwide (Shanghai, China). ZMT^®^ tablets were purchased from Xuhui Pharmaceutical Co., Ltd. (Chengdu, China).

### 2.2. Preparation of ZMT-OFDFs

The solvent casting method (SCM) was applied to prepare ZMT-OFDFs (Figure S1). Briefly, the polymeric materials, used at different weight ratios (PU = 300–500 mg; MDX = 0–100 mg), were dissolved in purified water (5 mL) and mixed for 2 h with a magnetic stirrer (RT 10 P, IKA, Königswinter, Germany) at 2000 rpm to obtain a homogenized solution (S1). Separately, ZMT (50 mg) and citric acid (48 mg) were dissolved in distilled water (5 mL) containing different plasticizer amount (15–25%) under continuous stirring for an additional 1 h at room temperature (RT) (S2). This drug-containing solution (S2) was added dropwise into the polymeric solution (S1) with continuous stirring and made up to a final volume of 10 mL. At the end, when the dispersion was found clear, requisite amounts of aspartame (24.3 mg) and mannitol (24.3 mg) were added in the preparation under mechanical stirring. The obtained transparent and homogenized solution was kept aside for 6 h to remove the entrapped air or bubbles. Finally, the solution was decanted into a 61 cm^2^ substrate, followed by drying at RT for 24 h. The resulting films were cautiously cut into 3 × 2 cm^2^ size, packed in an aluminum sachet, and stored in a desiccator until further assessment. The entire formulation process is schematically reported in Figure S2. 

### 2.3. Optimization of ZMT-OFDFs

To achieve optimized ZMT-OFDFs (Figure S5), a Box–Behnken experimental design with three factors and three levels was applied. The amount of PU (X_1_, 300–500 mg), MDX (X_2_, 0–100 mg), and PG (X_3_, 15–25%) were selected as independent variables. Cumulative release of ZMT at 5 min (Y_1_, %), D-time (Y_2_, s), and TS (Y_3_, MPa) were measured as responses (Table 1). The factors and variables were subjected to statistical analysis using Design-Expert software (trial version 10.0.3, Stat-Ease Inc., Minneapolis, MN, USA), that is specifically dedicated to performing design of experiments (DOE), to generate full-order polynomial equations and correlate the responses measured with the variables. ANOVA test was applied to evaluate the dependent variable’s influence on the studied responses at a 95% level of significance. All other ingredients, including citric acid, drug, aspartame, and mannitol, were kept constant to minimize experimental fluctuation. The optimized formulation design space was established targeting the quick dissolution, rapid disintegration, and favorable mechanical characteristics.

### 2.4. Thickness, Weight Uniformity, and Folding Endurance

The thickness of the film was measured by using a screw gauge (Q15A222169, Tresna Measuring Instrument Co., Ltd., Guilin, China) with a range of 0–25 mm and 0.001 mm resolution. The film (3 × 2 cm^2^) was kept between the spindles of the screw gauge and thickness measured at 5 different strategic points (the center and the four corners). The average ± SD was measured, and the mean value was calculated. 

The sample size of 3 × 2 cm^2^ was randomly selected from each batch and weighed on a digital analytical balance. The average weight was noted. 

The folding endurance was examined by repetitively folding each OFDFs at the same place until it broke, or once their integrity was lost. The result was obtained as the mean of three determinations.

### 2.5. Water Content, Moisture Absorption, and Surface pH

Three films from each batch were weighed on a digital analytical balance and kept in a hot air oven at 105 °C for 2 h. The dried films were weighted again, and the water content was calculated according to Equation (1):

Water content (%) = (initial weight − final weight)/(final weight) × 100(1)

Moisture content was assessed through the films-weight gain upon exposure to 79.5 ± 4% relative humidity in a desiccator at 25 ± 2 °C for 72 h, calculated by Equation (2):

Moisture content (%) = (final weight − initial weight)/(initial weight) × 100(2)

The film was dissolved in 5 mL of deionized water, and the pH was recorded with a digital pH meter (model: Vision Plus pH 6175, JENCO, San Diego, CA, USA) [27]. This study was repeated in triplicate.

### 2.6. Disintegration Time and Drug Content

Disintegration time was determined by placing a sample of dimension 3 × 2 cm^2^ in a petri dish containing about 25 mL of pH 6.8-simulated saliva. The dish was kept in a digital thermostatic oscillator (model: SHZ-82, Runhua Electric Co., Ltd., Suzhou, China) at 37 ± 0.5 °C, which was shaken continuously at 50 rpm. The time required for the disintegration of the film was noted. The test was triplicated.

Further, the drug content was measured by dissolving each film (of size 3 × 2 cm^2^) in 100 mL of pH 6.8-simulated saliva fluid through proper sonication. The solution was centrifuged at 10,000 rpm for 10 min, and subsequently analyzed by the given HPLC method.

### 2.7. Drug Dissolution

Films were examined for drug dissolution using a USP basket dissolution apparatus (RCZ-8-B, Shanghai, China) at a rotating speed of 100 rpm. The dissolution medium was 300 mL of pH 6.8-simulated saliva fluid maintained at 37.0 ± 0.5 °C. The specified aliquots were withdrawn at preset time intervals [28]. The supernatant was obtained by centrifugation of the sample at 10,000 rpm for 10 min. The Shimadzu HPLC system was then analyzed (model SPD-15c, Shimadzu Corporation, Kyoto, Japan) equipped with a UV-detector. A CST column (4.6 × 250 mm^2^, 5 μm) was applied as a stationary phase. The mobile phase was composed of 0.5% (*v*/*v*) triethylamine and acetonitrile (85:15, *v*/*v*, respectively). The mobile phase’s flow rate was set to 1.5 mL·min^−1^ with a column temperature set to 40 °C and a detection wavelength of 260 nm. 

### 2.8. Mechanical Characterization

Mechanical testing was conducted using a universal testing apparatus (model: Instron 3365 Dual Column Universal Testing System, Grass Valley, CA, USA) equipped with a 50 kg loaded cell. Each film with a dimension of 2 × 1 cm^2^ was fixed in tensile grips. The top grip stretched the sample at a rate of 100 mm·min^−1^ [29]. The TS and %E were calculated as the film broke according to Equations (3) and (4).

TS = (force at break)/(initial cross-sectional area of film)(3)

% E = (increase in length)/(original length) × 100(4)

### 2.9. Compatibility Study of Optimized Formulation

The differential scanning calorimetry (DSC) analysis was performed using the DSC instrument (model: TA 2010, TA Instruments, Inc, New Castle, DE, USA). Samples (approx. 3 mg) were heated in a hermetically sealed standard aluminum pan over a temperature range of 40–250 °C at a heating cycle of 20 °C·min^−1^ under a nitrogen atmosphere [30].

The X-ray diffractometry (XRD) study was carried out by XRD apparatus (model: Rigaku Mercury CCD, Tokyo, Japan) using Ni-filtered Cu K-alpha radiation (40 mA, 45 kV) at a range of 5–50°. The scanning temperature and time were set to 25 °C and 5 °C min^−1^, respectively [31].

### 2.10. Surface Morphology

The surface characteristics of ZMT powder and ZMT-OFDFs were examined by a field emission-scanning electron microscope (FE-SEM) (model: S-4700, Hitachi, Tokyo, Japan). Each sample was fixed on a metal stub using double-sided adhesive tape and was made electrically conductive by a colloidal gold at 10 mA in a vacuum prior to analysis. The SEM micrograph was recorded at an accelerated voltage of 10–15 kV under 1.5 K magnification.

### 2.11. Pharmacokinetics Study

Pharmacokinetic (PK) studies were carried under the animal care and use committee’s approval (Permit Number: SUDA20220407A02), Soochow University, China. Twelve male Sprague–Dawley rats (180–220 g, Shanghai, China) were randomly divided into two groups. Before the administration of ZMT OFDFs, 50 μL of deionized water was dropped into the oral cavity. The ZMT-OFDFs, at a dose of 10 mg·kg^−1^, were then cut into two pieces and placed upon the tongue of rats. An equivalent amount of ZMT was intra-gastrically administered as a control group. About 0.4 mL blood samples were collected in heparinized tubes via the retro-orbital plexus at 10, 30, 60, 120, 240, 480, and 720 min after drug administration. Samples were immediately centrifuged at 5000× *g* for 20 min. Plasma was collected and stored at −20 °C until further analysis. Up to 180 μL plasma was deproteinized with 1.8 mL dichloromethane by vortex mixing for 3 min [32]. After centrifugation at 10,000× *g* for 10 min at 4 °C, the supernatant organic layer was carefully transferred to a clean tube and dried using a light stream of nitrogen at 40 °C. The obtained residue was reconstituted with a 120 μL mobile phase and 10 μL rizatriptan (10 μg·mL^−1^) as an internal standard under vortex mixing for 3 min. The suspension was centrifuged at 10,000× *g* for 10 min, and 20 μL supernatant was injected into HPLC (Figures S6 and S7). The HPLC system and chromatographic column were similar as defined above. The slightly different wavelength and flow rate from the in vitro analysis was employed. The pharmacokinetic parameters were calculated using a non-compartmental assay (NCA) of the WinNonlin^®^ 6.1 pharmacokinetic software package (Certara, Princeton, NJ, USA).

### 2.12. Statistical Data Analysis

All experiments were conducted thrice, and the data are presented as a mean ± standard deviation (mean ± SD). The statistical differences between groups were performed by ANOVA using OriginPro 2019 (OriginLab Corporation, Northampton, MA, USA). Values of the in vivo parameters were statistically analyzed and compared amongst two groups by student *t*-test. The difference between mean of groups were reflected statistically significant and non-significant when *p* value < 0.05 and *p* > 0.05, respectively. 

## 3. Results and Discussion

### 3.1. Formulation Optimization

An SCM was employed for the development of ZMT-loaded OFDFs as it is the technique of choice for the formulation of OFDFs, as claimed by Cilurzo et al. [15]. MDX-based OFDs were prepared using both SCM and hot melt extrusion (HME) methods [15]. The results showed that OFDFs prepared with SCM had shorter in vitro and in vivo D-time compared to those prepared by the HME method. 

The Box–Behnken experimental model, designed by George E.P. and D. Behnken Box in the 1960s [33], is generally used because it is the most widely experimental design to construct higher-order surface prospects. A 13-run Box–Behnken design with three factors and three levels was preferred to prepare and optimize ZMT-OFDFs (Table 1). This compensates for fewer experiments (13 runs) compared to a full factorial design (27 runs) to maintain the higher-order surface response [34]. Such experimental design embroils the study of the effects of two or more independent factors and helps in studying the joint effect of the independent factors on an individual response. A three-factor, three-level design favored the construction of polynomial linear models and quadratic equations using the Design-Expert software (trial version 10.0.3, Stat-Ease Inc., Minneapolis, MN, USA). The different amounts of three independent variables, such as PU (X_1_), MDX (X_2_), and PG (X_3_), were designated based on the preliminary trials done before the experimental design is being applied. Accordingly, a mixture of PU:MDX with different weight ratios (3:0 to 5:1) was chosen for OFDFs to combine the advantages of the film-forming ability of PU and the high solubility property of the higher dextrose equivalent (DE) value of MDX. The values of all responses including cumulative release of ZMT at 5 min (Y_1_, %), D-time (Y_2_, s), and TS (Y_3_, MPa) were measured as responses which were fitted to the polynomial linear model. 

The effect of polymers and plasticizers concentration on the physicochemical properties (i.e., thickness, elongation, folding endurance, water content, moisture uptake) was assessed for ZMT-loaded OFDFs (Table 2).

Correlation coefficients (R^2^), lack of fit tests, and ANOVA tests were performed to validate the models (Table 3, Table 4, Table 5, Table 6, Table 7 and Table 8). ANOVA test was applied to describe the statistical significance of the model. The models showed a significant linear model for all responses, and their significance was confirmed via *p*-values < 0.05. F directed the regression equation’s resulting data and could clarify most of the variation in the responses. Each model was considered significant if *p* < 0.05, whereas *p* > 0.05 described a lack of fit for the corresponding responses. Moreover, the selected factors (X_1_ = PU, X_2_ = MDX, and X_3_ = PG) and their levels significantly (*p* < 0.05) impacted the designated responses (Y_1_ = release in 5 min, Y_2_ = D-time, and Y_3_ = TS) as presented in Table 1. It was concluded that X_1_ showed the most dominant antagonistic effect on response Y_1_, followed by the synergistic effect on response Y_2_ and Y_3_. The factors X_2_ and X_3_ showed a strong positive effect on Y_1_ and Y_2_ and a dominant-negative effect on Y_3_.

### 3.2. Feasibility and Appearance of OFDFs

In a preliminary study, preference was given to select the nature and concentration of the film-forming polymer and plasticizer. Initially, single PU was observed to develop OFDFs as a single polymer with optimal low concentration up to 300 mg (*w*/*w*). The resulting films were fragile with prolonged D-time. Sharma et al. and Kim et al. reported similar results and suggested that a composite polymer should be used instead [23,35]. Accordingly, MDX of higher DE value was incorporated into the PU film, which remarkably reduced the D-time, improved flexibility, and retained equilibrium moisture. These findings agree with the previous work of Elmeshad and Hagrasy [36]. In addition, ZMT-OFDFs composed of PEG were sticky and non-homogenized due to the immiscibility of PEG with MDX, that is consistent with Cilurzo et al. [15]. Films prepared with glycerol displayed a poor mechanical strength, which is attributed to their hygroscopic nature and tendency to retain moisture [14]. Hence, PG was chosen as a plasticizer for PU-MDX-based films as it produced easily detached and homogenous films and showed compatibility with all ingredients.

### 3.3. Thickness and Weight Uniformity

Table 2 displayed the effect of polymers and plasticizers concentration on the physicochemical properties of ZMT-loaded OFDFs. The mean thickness and weight of films were 25.5 ± 4.3 to 85.3 ± 6.5 μm and 22.2 ± 3.4 to 77.0 ± 4.2 mg, respectively. These parameters increased significantly (*p* < 0.05) with an increase in polymer amount irrespectively of polymer type (Figure S3). This is explained by the inclusion of solid ingredients that enhanced the total molecular volume of the films [23]. All formulations were non-sticky and were clear with uniform surface (Table S1). The drug content uniformity test demonstrated consistent dispersion of ZMT in all formulations following USP specifications. The surface pH of films was found in the pH range of 6 to 7, which suggests less potential to irritate oral mucosa and, hence, more tolerability to patients. The folding endurance of ZMT-OFDFs was significantly (*p* < 0.05) influenced by the plasticizer amount and varied from 76.0 ± 12.5 to 193.7 ± 12.6 folds. Films plasticized with 15% of PG had significantly (*p* < 0.05) lower folding endurance than 20% PG or 25% PG, when an equivalent proportion of polymer was used (Figure S4). The lower PG concentration produced an attraction force between the polymer-plasticizer, which was insufficient to overcome the hydrogen bonding forces between the polymer-plasticizer molecules.

### 3.4. Water Content (%) and Moisture Uptake (%)

**Figure 1:**
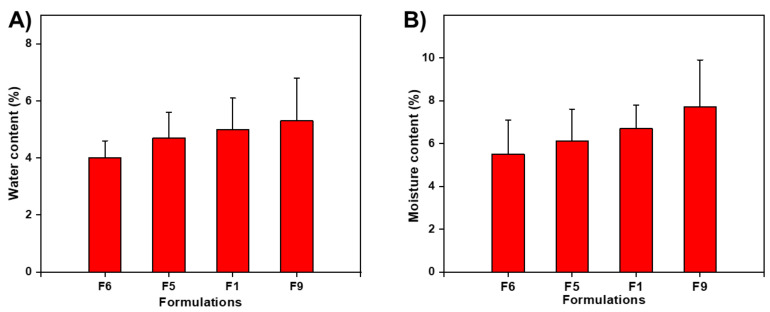
Effect of polymeric materials and plasticizer concentrations on (**A**) water content (%) of different ZMT-OFDFs and (**B**) moisture uptake (%) of different ZMT-OFDFs.

The presence of water contents in films delayed drying due to the plasticizing effect of water. Less moisture content caused brittleness, whereas higher water residue in OFDFs facilitated the adhesion. In our study, water content data (%) varied from 4.0 ± 0.6 to 6.9 ± 3.6% (Table 2). The water content (%) in the film tended to increase with increasing polymer and PG concentrations and vice versa. Figure 1A shows the effect of polymer ratio and plasticizer amount on water content (%) of ZMT-OFDFs. When the films were exposed to a relatively higher temperature (105 °C), then F6, comprising a low polymer concentration (300 mg), exhibited lower values of water content than F9, composed of PU:MDX (300:100 mg). This might be due to the hydrophilic nature of polymers. Moreover, an increase in PG content in the composite films resulted in a proportional increase in water content. ZMT-OFDFs with 15% plasticizer (F5) showed remarkably lower water content values than plasticized with 25% (F1), as shown in Figure 1. These results are similar to those achieved by Jantrawut et al. [37]. This response might be due to the hydrophilic nature of PG and might produce a sizeable hydrodynamic complex of polymer-plasticizer and water.

On the other hand, moisture uptake (%) provides basic information about OFDFs’ stability. The increase in water permeability with increasing hydrophilic polymers and plasticizer concentration is expected in OFDFs [38]. In this study, the moisture uptake (%) of ZMT-OFDFs was found in the range of 4.6 ± 1.4 to 7.7 ± 2.2%, as shown in Table 2. Moreover, the concentration of MDX and PG remarkably enhanced the moisture uptake. Figure 1B shows ZMTs-OFDFs prepared with 300 mg PU only (F6) were lower than ZMT-OFDFs prepared with PU:MDX in a ratio of 300:100 mg (F9), when the same amount of PG was used. In addition, alterations in plasticizer content also influenced moisture absorption (%). ZMT-OFDFs plasticized with 15% PG (F5) absorbed less moisture than films composed of 25% PG (F1) when an equivalent polymer amount was used. The overall trend of upsurge in moisture uptake (%) with the increase in both MDX and PG concentration was noticed. This is due to the enhancement in MDX mobility, with the PG getting between the polymer chains, thereby divulging more of its strands for moisture uptake [36]. On the other hand, ZMT-OFDFs prepared with only PU, as a polymer matrix, absorbed minimum moisture content (Figure 1B). It is attributed to straight polysaccharide chain of PU, which lacks side chains. As a result, the molecular chains in the OFDFs were closely aligned, therefore, the penetration of moisture molecules was difficult to pass through PU [39]. Thus, the incorporation of MDX to PU is an effective and efficient method of preventing brittleness of ZMT-OFDFs.

### 3.5. In Vitro Drug Release Study

**Figure 2:**
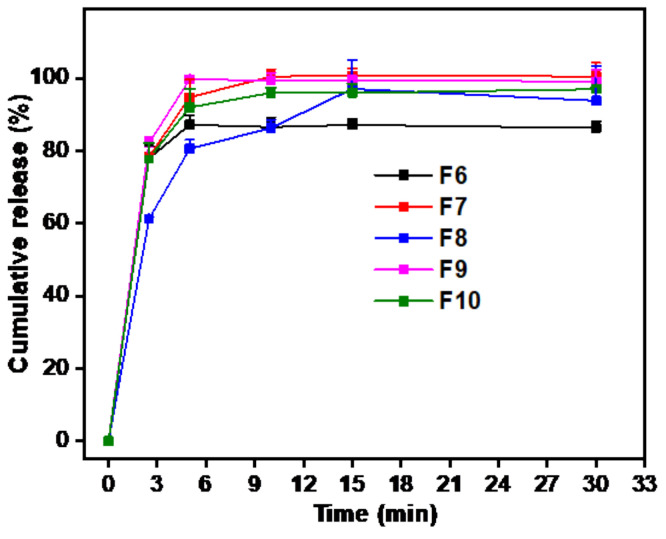
In vitro release profile of ZMT from OFDFs in simulated saliva fluid, at pH 6.8. Values are plotted as mean ± S.D. (*n* = 3).

The in vitro release performance of ZMT-loaded OFDFs was evaluated in pH 6.8-simulated saliva, as shown in Figure 2. When an equivalent amount of plasticizer (20%) was used, the release (%) of ZMT-OFDFs (t = 15 min) composed of 300 mg PU (F6) was remarkably faster than the prepared one containing 500 mg PU (F8). This may be due to the wicking effect caused by the high polymer concentration. It is thought that this produces a thicker barrier layer, which impedes the moisture penetration leading to prolonged D-time and dissolution. The ZMT-OFDFs composed of low polymer concentration dissolve and form porous channels more easily, which is beneficial for the rapid disintegration and drug release [40,41,42]. All the herein tested ZMT-OFDFs released ZMT completely at 15 min. The release curve difference was observed at the early time points (2 min, 5 min), as shown in Table 1. The dissolution performance of ZMT-OFDFs with 100 mg MDX (F9) was significantly (*p* < 0.05) superior to that without MDX (F6) in the first 5 min. This might be due to the effect of oligosaccharide MDX on PU polymer structure, which enhances the water permeability and causes a rapid disintegration of ZMT-OFDFs while increasing the solubility and diffusion of the drug [36]. In addition, the alteration in the PG amount impacted the ZMT-OFDFs drug release mechanism. When ZMT-OFDFs were prepared with the same polymer proportion (PU:MDX, 400:100), the release rate of ZMT-OFDFs (t = 15 min) plasticized with 25% PG (F7) was slightly faster compared to that one prepared with 15% PG (F10). This might be because the polar (-OH) group generated a plasticizer-polymer hydrogen bond, substituting the polymer–polymer interaction in biopolymer ZMT-OFDFs, resulting in a more porous and less dense polymer structure that can easily disrupt at weak force, thereby ensuring fast disintegration and dissolution.

The software recommended a significant linear model for response release at 5 min, and their lower SD values demonstrated fewer differences in the suggested model (Table 3). Relatively higher polynomial coefficient (R^2^ = 0.96) values of response released at 5 min guaranteed high prognostic propensity. Similarly, the suggested linear model explained variability around the mean; thus, the applied model could elucidate about 96% of the variability in the results. Consequently, the model was substantiated for full model analysis of variance (ANOVA), as shown in Table 4.

The best model F value for Y_1_ (71.4) and regression coefficients having a *p* < 0.05 value indicated the model’s significance. The dependent variable release (Y1) was strongly dependent on the corresponding factors (X_1_ = PU, X_2_ = MDX, and X_3_ = PG). The ANOVA results were used to generate a statistical model that specified a reasonable covenant among dependent and independent variables. Equation (5) was generated when drug release (Y_1_) was correlated with independent variables (X_1_, X_2_, and X_3_):

Y_1_ = 89.15 − 4.96X_1_ + 4.69X_2_ + 1.31X_3_ (5)

It can be seen from Equation (5) that X_1_ (PU amount) signified a synergistic influence on Y_1,_ whereas X_2_ (MDX amount) and X_3_ (PG amount) had an antagonistic impact. This means that even with slight increases in PU concentration in ZMT-OFDFs, drug release was significantly decreased. Following on from this, the negative sign indicates that as the amount of MDX and PG increased, the drug release of ZMT-OFDFs was dramatically enhanced. From the above equation, it is evident that the impact of X_2_ on the response (Y_1_) was significant (*p* < 0.05) compared to that of X_3_. The findings are following the suggested D-time results of ZMT-OFDFs. 

**Figure 3:**
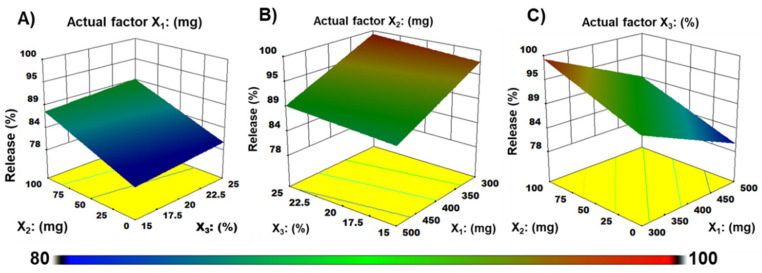
Three-dimensional (3-D) plots demonstrate the effect of (**A**) Pullulan (X1), (**B**) Maltodextrin (X2), (**C**) Propylene glycol (X3) on response release at 5 min (Y1) of ZMT-OFDFs.

Figure 3 shows the correlation between the dependent variables and response release at 5 min. It can be seen from Figure 3A that the drug release at 5 min (Y_1_) decreased as the amount of PU (X_1_) increased, while it increased as the ratio of X_2_ and X_3_ in ZMT-OFDFs increased. It was clarified that at fixed actual factor of X_1_ (PU = 500 mg), with any level of X_2_ (MDX = 0–100 mg), and X_3_ (PG = 15–20%) demonstrated 82.2 to 87.9% of drug release. In addition, a sharp rate of early release was observed when the X_2_ amount was maintained at a high level (MDX = 100 mg), X_1_ at a low level (PU = 300 mg), at a condition that X_3_ increased to medial level (PG = 20%). A fixed factor of X_3_ in Figure 3C explained the significant antagonistic effect of X_1_ and synergistic effects of X_2_ on the release (%) of ZMT from OFDFs. The results agree with the software-generated linear equation of drug release at 5 min.

### 3.6. Disintegration Time

When ZMT-OFDFs were plasticized with the same amount of plasticizer (20%), the D-time of films comprised with PU:MDX:PG = 300:100:20 (F9) disintegrated faster than PU:MDX:PG = 500:100:20 (F11) due to the wicking disintegration mechanism [40,41], as shown in Figure S3. On the other hand, the D-time of ZMT-OFDFs (F9) composed of PU:MDX = 300:100 was less than PU 300 (F6, excluding MDX) as shown in Table 1. This could be attributed to the hydrophilic/oligosaccharide nature of MDX that influenced the polymer chains attrition which primarily enhanced the water penetration to films, leading to its fast disintegration. The results agree with the previous work of El Meshad and El Hagrasy [36]. Moreover, when using the same proportion of polymer (PU:MDX = 400:100), the films comprised of 25% PG (F7) disintegrated faster than those plasticized with 15% PG (F10). This might be attributed to enough PG that disrupted the polymer chain after exposure to water, which ultimately breaks the small polymer crystallites, leading to faster disintegrating of the film. The estimated regression coefficient of the entire model is tabulated in Table 5. The obtained R^2^ value of the whole model was 0.946 indicating the significance of the model. It means that the model could describe around 94.6% of variability around the mean. ANOVA of the entire model is shown in Table 6. Model F value of 180.4 indicated that the proposed model was significant. The regression output demonstrated a *p* < 0.05 value specifying the significance of the model.

The following multiple linear regression equation (Equation (6)) prevailed from the model for the response D-time (Y_2_).

Y_2_ = 29.89 + 9.68X_1_ − 3.48X_2_ − 1.46X_3_ (6)

A positive sign in Equation (6) demonstrated that the independent variables positively affected the response variable. A negative sign indicated that the independent variables were negatively related to the response variable. The results revealed that with the increase of X_1_ (PU amount), the D-time of ZMT-OFDFs was prolonged, while X_2_ (MDX amount) and X_3_ (PG%) shortened the D-time. The order of impact was X_1_, X_2_, and X_3_.

**Figure 4:**
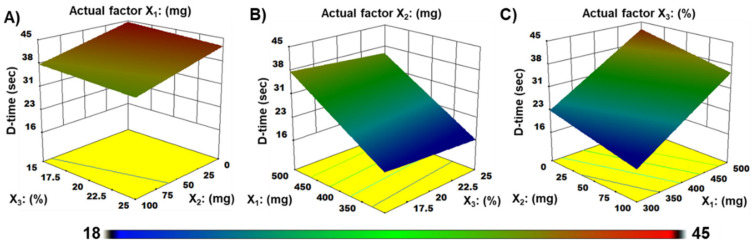
Three-dimensional (3-D) plots demonstrate the effect of (**A**) pullulan (X_1_), (**B**) maltodextrin (X_2_), (**C**) propylene glycol (X_3_), on D-time (Y_2_) of ZMT-OFDFs.

The contour plot and three-dimensional (3D) response surface model showing the effect of pullulan (X_1_), maltodextrin (X_2_), or propylene glycol (X_3_), on D-time (Y_2_) of ZMT-OFDFs, are presented in Figure 4. It is perceived from the plots that ZMT-OFDFs composed of a high level of X_1_ (PU = 500), with any level of X_2_ (MDX = 0–100 mg) and X3 (PG = 0–25%) displayed a D-time that varied from 36.2 to 44.5 s (Figure 4A). Films prepared with a fixed amount of X_2_ (MDX = 100 mg), at a condition where X_1_ is increasing from a low to a high level, increased the D-time of films significantly (*p* < 0.05). An increase in the amount of X_3_ slightly decreased the D-time of ZMT-OFDFs, as shown in Figure 4B. However, a film comprised of a low level of X_1_ (PU = 300 mg), a high level of X_2_ (MDX = 100 mg), and a medium level of X_3_ (PG = 20%) displayed a significant depression in D-time to 18.3 s, as shown in Figure 4C.

### 3.7. Mechanical Properties Analysis

**Figure 5:**
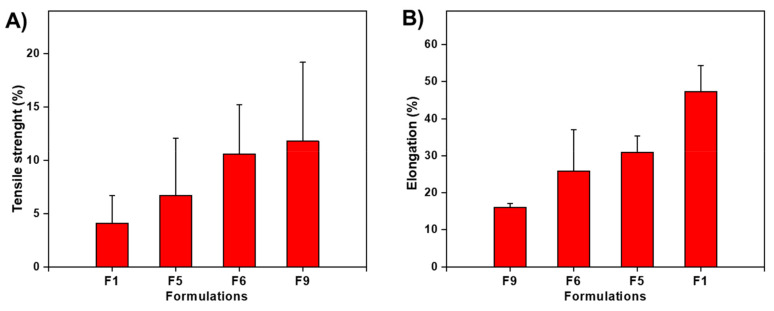
Impact of maltodextrin and propylene glycol concentrations on mechanical properties of ZMT-OFDF formulations F1, F5, F6, and F9. (**A**) Tensile strength (%); (**B**) Elongation (%). Data are expressed as mean ± SD (*n* = 3).

The mechanical results showed that as the amount of MDX and PG content increased, the TS of ZMT-OFDFs decreased, and the %E increased (Table 1). When ZMT-OFDFs were plasticized with the same amount of PG, F9 (PU:MDX = 300:100) had lower TS and higher %E than F6 (PU = 300), as shown in Figure 5. Moreover, the ZMT-OFDFs prepared with PU (single) showed higher TS and brittleness when exposed to a dry environment. The results agree with the previous work of Sharma et al. [35] and Kim et al. [23]. The incorporated MDX with a high hydrolysis conversion rate (DE value) exerted the combined effect of PU-MDX in OFDFs which improved the mechanical properties of ZMT-OFDFs. This can be explained by the different bonding systems between PU-MDX. The PU-MDX leading chains segmental flexibility is related to the α-1, 6, and α-1, 4 bonds in the structure. The first one is relatively rigid, and the latter has a larger flexibility region [42]. On the other hand, as the PG content increased, the TS of ZMT-OFDFs decreased significantly, while the extensibility increased. At the same proportion of polymer, the film composed of 25% PG (F1) had lower TS and higher %E than 15% PG (F5), as shown in Figure 5. This may be due to the easy insertion of low molecular weight and highly hydrophilic PG into the polymer chain; this resulted in a hindered association between the PU-MDX chains, in the meantime increasing the molecular mobility of the polymer chain, enhancing the elasticity and reducing the rigidity of the ZMT-OFDFs [43].

The polynomial equation of the full model for TS is presented in Table 7. The R^2^ value was found to be 0.940, indicating the model significance and defining 94.0% of variability around the mean. ANOVA of the whole model is shown in Table 8. The model F value of 70.6 suggested that the model was significant. Furthermore, *p* < 0.05 value indicated that the model terms were substantial.

The multiple linear regression for response TS (Y_3_) was expressed by Equation (7), as follows:

Y_3_ = 8.42 + 1.91X_1_ − 1.70X_2_ − 6.69X_3_ (7)

Equation (7) shows that X_1_ (PU amount) had a positive effect on TS, which means that as the X_1_ amount increased, the TS of ZMT-OFDFs increased. The negative results of X_2_ and X_3_ on TS indicate that as these factors’ concentration increased, the TS decreased. The order of influence of independent variables on response TS was X_3_ > X_2_ > X_1_.

**Figure 6:**
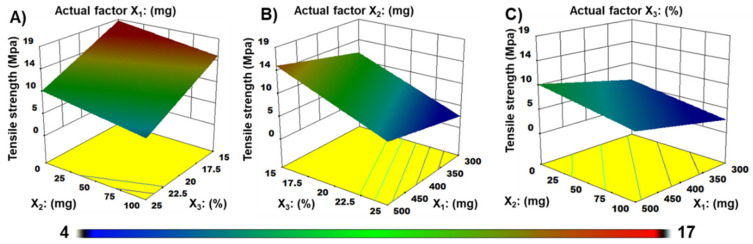
Three-dimensional (3-D) plots demonstrate the effect of (**A**) pullulan (X_1_), (**B**) maltodextrin (X_2_), (**C**) propylene glycol (X_3_) on tensile strength (Y_3_) of ZMT-OFDFs.

Three-dimensional surface plots explored the highest TS of about 17.1 MPa occurred roughly at the lower level of PG and MDX (Figure 6A). At a fixed actual factor X_2_, the TS became lower with increasing PG amount to PU in films (Figure 6B). Contour plots in Figure 6C revealed that PG, at the highest level of 25% to any polymer ratio, significantly reduced the TS of ZMT-OFDFs.

### 3.8. Validation of Optimum Formulation

The Design-Expert (DE) software analyzed all responses in one measurement to predict the optimum level of independent variables based on the desirability function. The model-generated optimized ZMT-OFDFs (F9) was composed of PU (300 mg), MDX (100 mg), and PG (20%), respectively. The films were evaluated for drug release (Y_1_, %), D-time (Y_2_, s), and TS (Y_3_, MPa) to prove the validity of the model (Table 9). The values were calculated based on derived polynomial equations compared with predicted values to confirm their adequacy and reliability by the given equation [14].

(8) $$\mathrm{Relative}\;\mathrm{error}\;\left(\%\right)=1+\frac{\mathrm{predicted}\;\mathrm{value}-\mathrm{experimental}\;\mathrm{value}}{\mathrm{predicted}\;\mathrm{value}\;}\times 100$$

Table 9 displayed the experimental and predicted testing of the optimized formulation. Experimental results were closed to those predicted by the model. The difference between the actual and the model predicted properties were statistically insignificant (*p* > 0.05), which ensured model validity.

### 3.9. Compatibility Analysis

**Figure 7:**
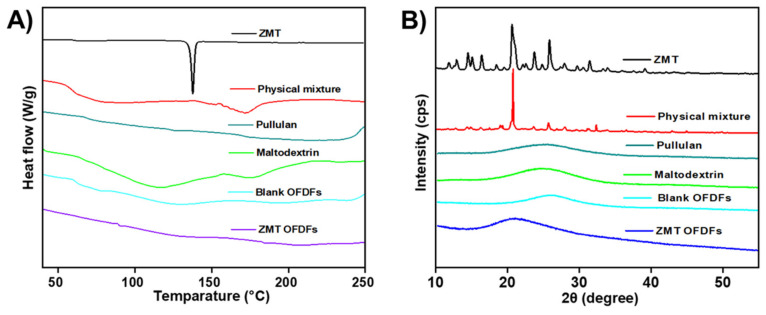
Compatibility analysis. (**A**) DSC thermograms, (**B**) XRD spectrum of ZMT, physical mixture of polymers and drug, Maltodextrin, Pullulan, Blank OFDFs, and ZMT-OFDFs.

The DSC study was performed to detect any potential interaction amongst pure drug and emerged polymers in ZMT-OFDFs (Figure 7). Their corresponding irreconcilability was illustrated by the shifting, arrival, or disappearance of melting peaks. The DSC thermogram of the pure ZMT displayed an endothermic transition at 140 °C indicating that ZMT melts at a temperature of 140 °C, as shown in Figure 7A [7]. The disappearance of peaks in the thermogram of ZMT-OFDFs signified complete molecular miscibility and uniform dispersion of ZMT in film-forming components. Additionally, the absence of extra peaks indicated the lack of ZMT recrystallization in the ZMT-OFDFs [41].

An XRD study was carried out to verify the crystallinity transformation of a model drug in the optimized formulation. Figure 7B shows the patterns of pure ZMT, physical mixture of drug and polymer, MDX, PU, blank OFDFs, and optimized ZMT-OFDFs. The XRD diffractogram of the pure ZMT exhibited intense and sharp peaks at 11.4, 12.4, 13.6, 15.8, 19.5, 22.1, 23.8, and 29.1° that indicated the pure crystalline form of ZMT [6]. The characteristic crystalline peaks of ZMT were detected in the physical mixture of drug and polymers. The patterns of MDX, PU, and blank OFDFs displayed a broad peak at 18.1° which specified polymers’ amorphous form. The hallo diffractograms of ZMT-OFDFs showed a broad rise at 16.3°, respectively. The results indicated that the crystalline state of ZMT had been partially transformed into an amorphous form. It might be attributed to the fact that drug crystallinity could be converted to an amorphous form during chemical manipulation [35,41].

### 3.10. Surface Morphology Analysis

**Figure 8:**
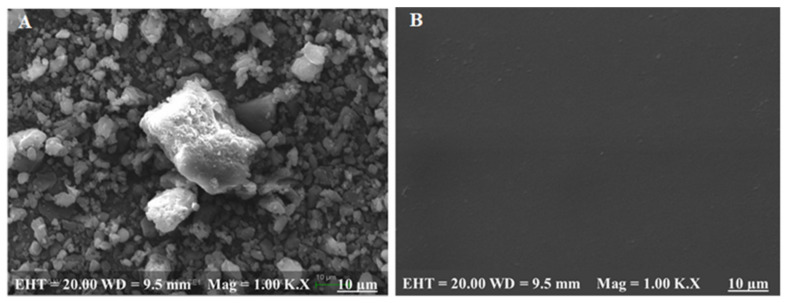
Scanning electron microscopy (SEM) of (**A**) ZMT powder, (**B**) optimized formulation.

The SEM image of ZMT powder demonstrated a stable crystal structure, as shown in Figure 8A. Whereas the SEM image of ZMT-OFDFs displayed a smooth surface without any scratches or traverse striations on the surface (Figure 8B). The results indicated the proper miscibility and uniform distribution of ZMT in the OFDFs [14,44].

### 3.11. Pharmacokinetics Study

**Figure 9:**
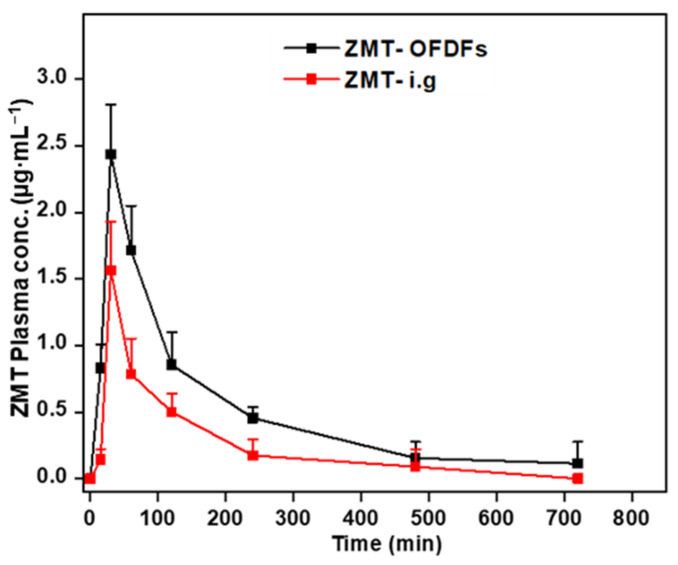
Pharmacokinetics profile of ZMT after oral administration of OFDFs and intragastric suspension at a dose of 10 mg·kg^−1^ to rats. Values are given as mean ± S.D (*n* = 6).

The plasma concentration profile of ZMT-OFDFs and ZMT intragastric suspension in rat plasma was plotted against time (Figure 9). The peak plasma concentration (*C*_max_), the time to reach the maximum peak (*T*_max_), the area under the drug time curve (AUC_0–t_), and the mean retention period (MRT) were determined (Table 10).

The corresponding PK parameters of both dosage forms were different from each other. The C_max_ of ZMT-OFDFs and ZMT intragastric suspensions were 2.44 ± 0.34 μg·mL^−1^ and 1.56 ± 0.37 μg·mL^−1^, respectively, with significant difference (*p* < 0.05), reaching the peak at 0.5 h. The AUC_0–t_ among the two groups was 5.89 ± 0.94 μg·h·mL^−1^ and 2.82 ± 1.02 μg·h·mL^−1^, with significant differences (*p* < 0.05). The MRT of the two formulations was 2.9 ± 0.7 h and 2.3 ± 0.9 h, respectively, indicating that the retention time of ZMT in vivo was nearly similar. The obtained results are consistent with the earlier work of Singh et al. and Bhagawati et al. [44,45]. The above results show improved absorption of ZMT-OFDFs compared to ZMT intragastric suspensions in the rats. This can be attributed to the faster disintegration and dissolution of OFDFs leading to rapid absorption of ZMT from the oral mucosa which undoubtedly resulted in a decreased pre-systemic biotransformation and degradation of the digestive tract environment [16].

## 4. Conclusions

In this study, ZMT-OFDFs were successfully prepared by SCM based on the Box–Behnken design. Considering the feasibility of ZMT-OFDFs formulation, an optimized PU-MDX mixture was used as polymeric materials and PG as a plasticizer. The formulated OFDFs were transparent with smooth surface without any conceivable interactions between the model drug and polymers. The multiple regression exploration of the outcomes led to equations that pronounce adequately the effect of the selected independent variables on the responses under current study. The desirability function directed to the optimum values of the selected factors at which the formulated OFDFs showed fast drug release, rapid D-time, and favorable mechanical possessions. The in vitro and in vivo results of ZMT-OFDFs showed that the fabrication of ZMT-OFDFs had some advantages over the traditional oral administration, such as sample preparation method, convenient administration, fast disintegration, and improved patient compliance. In addition, ZMT-OFDFs could offer a solution to some of the challenges that exist in the current migraine therapy. Impaired absorption of ZMT is a significant problem considering high reports of gastrointestinal (GIT) dysfunction as a symptom of migraine. ZMT-OFDFs bypass this by directly entering to the systemic circulation with reduced hepatic first-pass effect. The preliminary findings indicated that ZMT-OFDFs prepared with 3:1 (PU to MDX) and 20–25% PG content (based on solid content) is the optimal formulation that meets the quality requirements of rapid disintegration, dissolution, and favorable mechanical properties for the oral cavity.

This study is expected to provide a new basis for the research and development of anti-migraine drugs.

## Figures and Tables

**Table 1:** Box–Behnken DOE of independent variables (X_1_, X_2_, and X_3_) and their influences on responses (Y_1_, Y_2_, and Y_3_). All values are expressed as mean ± S.D (*n* = 3).

Film Code	Pullulan (mg, X_1_)	Maltodextrin (mg, X_2_)	Propylene Glycol (%, X_3_)	Release at 5 min (Y_1_, %)	D-Time (Y_2_, s)	Tensile Strength (Y_3_, MPa)
F1	300	50	25	94.3 ± 1.7	20.5 ± 1.9	4.1 ± 2.6
F2	400	50	20	90.3 ± 1.5	28.2 ± 3.7	10.4 ± 2.0
F3	400	0	25	85.2 ± 4.2	31.7 ± 2.7	8.6 ± 8.1
F4	500	50	25	82.9 ± 1.3	40.2 ± 3.2	7.6 ± 3.5
F5	300	50	15	92.0 ± 5.2	22.2 ± 2.3	11.8 ± 7.4
F6	300	0	20	87.2 ± 2.5	24.7 ± 1.4	10.6 ± 4.6
F7	400	100	25	94.8 ± 4.8	25.2 ± 3.5	5.0 ± 4.0
F8	500	0	20	80.6 ± 2.4	44.5 ± 3.5	14.6 ± 2.1
F9	300	100	20	99.8 ± 0.5	18.3 ± 1.9	6.7 ± 5.4
F10	400	100	15	92.0 ± 5.2	27.2 ± 3.1	15.7 ± 0.4
F11	500	100	20	87.9 ± 3.0	36.2 ± 3.7	9.9 ± 6.5
F12	500	50	15	82.2 ± 1.0	42.2 ± 5.9	16.4 ± 1.1
F13	400	0	15	84.0 ± 1.2	33.8 ± 3.1	17.1 ± 2.0

Amounts of other ingredients, such as aspartame, mannitol, drug, and distilled water (10 mL) were kept constant.

**Table 2:** Results of physical evaluation parameters of ZMT-OFDFs. All values are expressed as mean ± S.D (*n* = 3).

Film Code	Thickness (μm)	Elongation (%)	Folding Endurance (Folds)	Water Content (%)	Moisture Uptake (%)
F1	33.9 ± 5.1	47.3 ± 7.0	129.0 ± 11	5.0 ± 1.1	6.7 ± 1.6
F2	56.8 ± 6.8	26.2 ± 0.8	100.3 ± 9.5	5.5 ± 1.9	6.0 ± 1.5
F3	40.1 ± 5.2	27.7 ± 1.5	142.3 ± 16.5	5.0 ± 1.4	5.6 ± 1.3
F4	75.1 ± 6.3	39.2 ± 8.4	193.7 ± 12.6	6.3 ± 2.8	5.1 ± 2.1
F5	36.7 ± 4.9	16.1 ± 1.0	76.0 ± 12.5	4.7 ± 1.5	6.1 ± 1.5
F6	25.5 ± 4.3	25.8 ± 11.3	121.7 ± 8.5	4.0 ± 0.6	5.5 ± 0.8
F7	64.6 ± 6.3	53.2 ± 12.4	108.3 ± 8.0	6.0 ± 2.4	6.8 ± 2.6
F8	66.4 ± 5.5	18.0 ± 0.3	178.7 ± 11	5.5 ± 2.4	6.5 ± 3.0
F9	44.1 ± 5.8	30.9 ± 4.4	109.3 ± 9.5	5.3 ± 1.5	7.7 ± 2.2
F10	62.0 ± 7.4	13.9 ± 0.2	88.7 ± 12.9	6.4 ± 2.6	6.4 ± 2.6
F11	85.3 ± 6.5	24.0 ± 1.1	146.3 ± 7.4	6.9 ± 3.6	5.5 ± 3.0
F12	73.2 ± 8.7	10.5 ± 3.9	103.3 ± 7.8	6.0 ± 2.9	4.8 ± 2.4
F13	44.9 ± 6.1	11.8 ± 0.9	96.3 ± 9.6	4.9 ± 1.4	4.6 ± 1.4

**Table 3:** Model summary and statistics of drug release at 5 min Y_1_ response.

Responses	Model	Std. Dev.	R^2^	Adjusted R^2^	Predicted R^2^	Press	Statistical Analysis
Drug release at 5 min (Y_1_)	Linear	1.33	0.960	0.946	0.918	32.55	*
	2FI	1.13	0.981	0.961	0.920	31.43	
	Quadratic	0.75	0.996	0.983		+	
	Cubic					+	#

* *p* < 0.05 and ^#^ *p* > 0.05 indicated indicated statistical significance and insignificance, respectively.

**Table 4:** Summary of ANOVA for drug release at 5 min Y_1_ response.

Responses	Source	Sum of Squares	d.f.	Mean Square	F Value	*p*-Value Prob > F	Statistical Analysis
Drug release at 5 min (Y_1_)	Model	378.9	3	126.3	71.4	<0.0001	**
	X_1_	197.0	1	197.0	111.3	<0.0001	**
	X_2_	175.8	1	175.8	99.3	<0.0001	**
	X_3_	6.1	1	6.1	3.5	0.0958	#

** *p* < 0.0001 and ^#^ *p* > 0.05 indicated statistical significance and insignificance, respectively.

**Table 5:** Model summary and statistics of D-Time (Y_2_) response.

Responses	Model	Std. Dev.	R^2^	Adjusted R^2^	Predicted R^2^	Press	Statistical Analysis
Disintegration (Y_2_)	Linear	1.26	0.984	0.9782	0.9695	26.42	*
	2FI	1.49	0.985	0.9694	0.9387	53.17	
	Quadratic	0.53	0.999	0.9961		+	*
	Cubic					+	#

* *p* < 0.05 and ^#^ *p* > 0.05 indicated statistical significance and insignificance, respectively.

**Table 6:** Summary of ANOVA for D-time (Y_2_) response.

Responses	Source	Sum of Squares	d.f.	Mean Square	F Value	*p*-Value Prob > F	Significant/ Non-Significant
Disintegration (Y_2_)	Model	853.1	3	284.4	180.4	<0.0001	**
	X_1_	748.8	1	748.8	475.0	<0.0001	
	X_2_	96.6	1	96.6	61.3	<0.0001	
	X_3_	7.6	1	7.6	4.8	<0.0557	

** *p* < 0.0001 indicated statistical significance.

**Table 7:** Model summary and statistics of tensile strength (Y_3_) response.

Responses	Model	Std. Dev.	R^2^	Adjusted R^2^	Predicted R^2^	Press	Statistical Analysis
Tensile strength (Y_3_)	Linear	1.05	0.955	0.9401	0.90	21.92	**
	2FI	1.18	0.963	0.9252	0.79	46.01	
	Quadratic	0.94	0.988	0.9525		+	
	Cubic					+	#

** *p* < 0.0001 and ^#^ *p* > 0.05 indicated statistical significance and insignificance, respectively.

**Table 8:** Model summary and statistics of tensile strength (Y_3_) response.

Responses	Source	Sum of Squares	d.f.	Mean Square	F Value	*p*-Value Prob > F	Significant/ Non-Significant
Tensile strength (Y_3_)	Model	211.7	3	70.6	63.8	<0.0001	**
	X_1_	29.3	1	29.3	26.4	<0.0006	
	X_2_	23.1	1	23.1	20.9	<0.0013	
	X_3_	159.3	1	159.3	144.0	<0.0001	

** *p* < 0.0001 indicated statistically significance.

**Table 9:** Difference between actual and predicted values of optimized ZMT-OFDFs.

PU:MDX:PG = X_1_:X_2_ (mg):X_3_ (%)	Response Variables	Actual Values	Predicted Values	Relative Error (%)
	Y_1_ (Release)	99.8	98.4	−1.5
PU:MDX:PG 300 mg:100 mg:20%	Y_2_ (D-time)	18.3	17.2	−6.2
	Y_3_ (Tensile strength)	6.7	7.0	4.8

**Table 10:** Pharmacokinetics profile of ZMT after oral administration of OFDFs and intragastric suspension at a dose of 10 mg·kg^−1^ to rats.

No.	PK-Parameters	ZMT-OFDFs	ZMT-ig Suspension
1	C_max_ (μg·mL^−1^)	2.44 ± 0.34	1.56 ± 0.37
2	T_max_ (h)	0.5	0.5
3	AUC (0–t) (ng·h·mL^−1^)	5.89 ± 0.94	2.82 ± 1.02
4	MRT (h)	2.9 ± 0.7	2.3 ± 0.9

Values are expressed as mean ± S.D., *n* = 6.

## Data Availability

Data sharing not applicable.

## Supplementary Materials

The following supporting information can be downloaded at: https://www.mdpi.com/article/10.3390/ma15103591/s1, Figure S1: Macrograph of the ZMT-loaded OFDFs; Figure S2: Schematic representation of blood sample treatment for pharmacokinetics studies using the rat model; Figure S3: Effect of polymer concentrations on thickness of film; Figure S4: Effect of plasticizer concentration on folding endurance; Figure S5: Effect of polymer concentrations on D-time; Figure S6: Typical chromatograms of blank solution (A), 10 μg·mL^−1^ standard solution (B), sample obtained after dissolution of ZMT-OFDFs (F1) at 5 min (C), typical calibration curve of ZMT in simulated saliva at pH 6.8 for in-vitro studies (D); Figure S7: Representative chromatogram of blank rat plasma (A), plasma spiked with 10 μg·mL^−1^ ZMT (B), plasma spiked with 10 μg·mL^−1^ RZT (C), plasma spiked with ZMT-RZT (D), plasma sample taken at 0.5 h after oral administration of ZMT-OFDFs to rats (E), and a selected calibration curve of ZMT for in-vivo studies (F). Table S1: Feasibility, pH, and drug content (%) determination of ZMT-OFDFs.
