# Supplementary material for: Development and Characterizations of Pullulan and Maltodextrin-Based Oral Fast-Dissolving Films Employing a Box–Behnken Experimental Design
Source: Materials (Basel). 2022 May 18;15(10):3591. doi: 10.3390/ma15103591 (PMC9146677; doi:10.3390/ma15103591)
Supplement: Supplementary file 1 [file materials-15-03591-s001.zip › materials-1663978-supplementary.pdf]

materials

Supplementary Information

Development and Characterizations of Pullulan and Maltodextrin-Based Oral Fast-Dissolving Films Employing a Box–Behnken Experimental Design

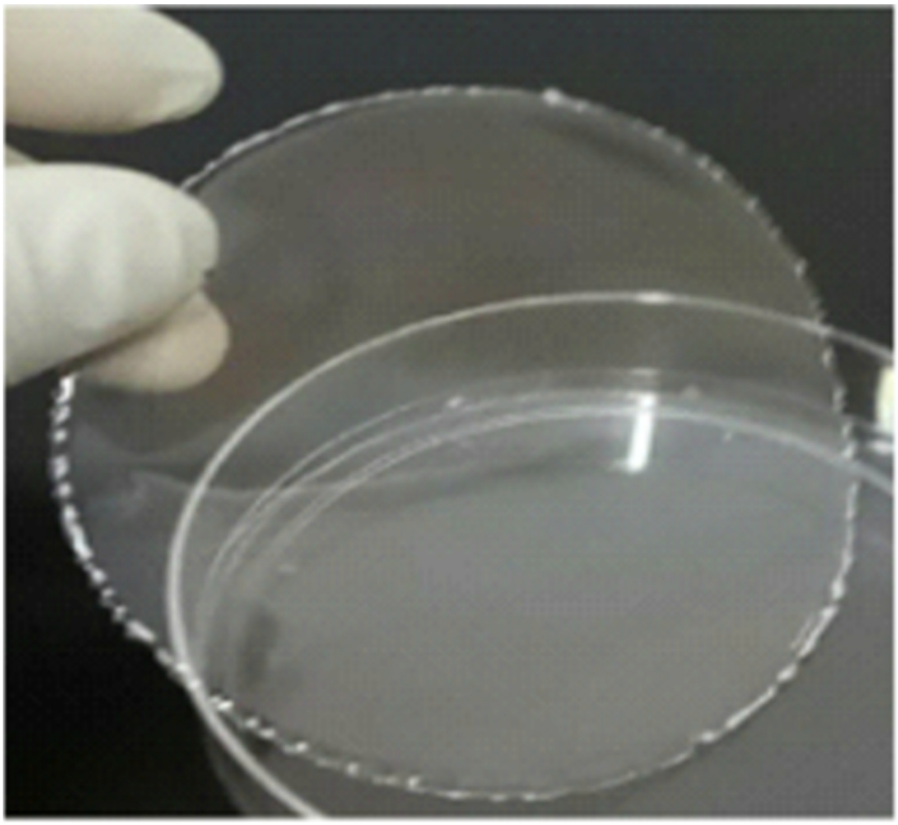

Figure S1. Macrograph of the ZMT-loaded OFDFs.

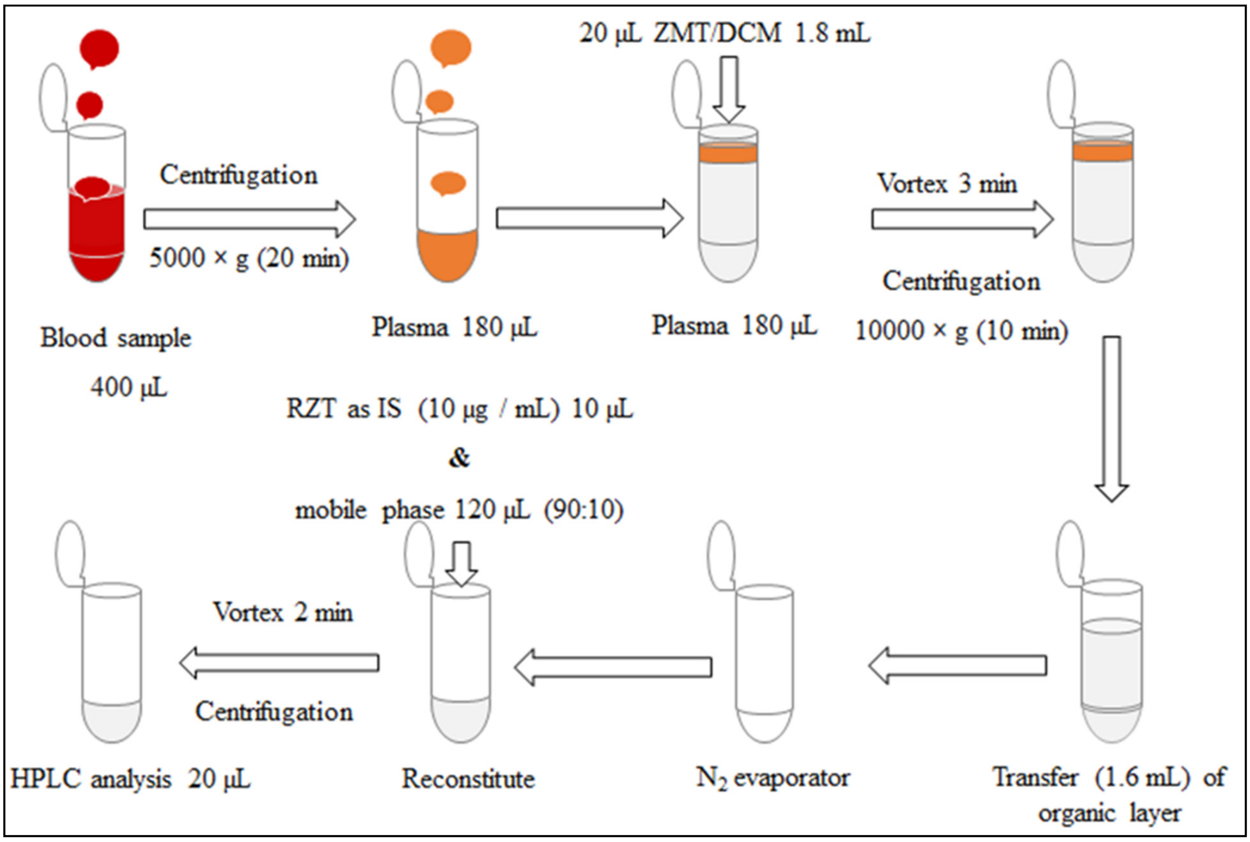

Figure S2. Schematic representation of blood sample treatment for pharmacokinetics studies using the rat model.

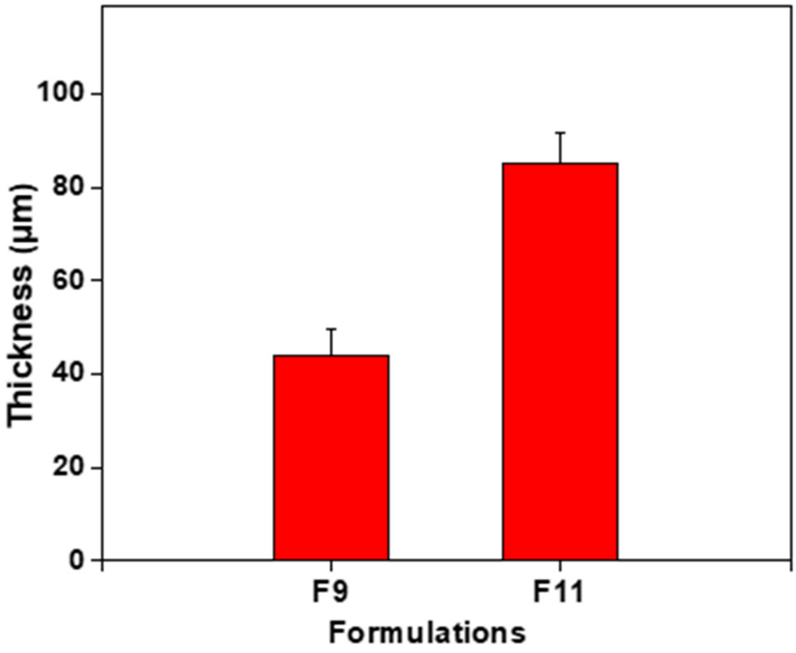

Figure S3. Effect of polymer concentrations on thickness of film.

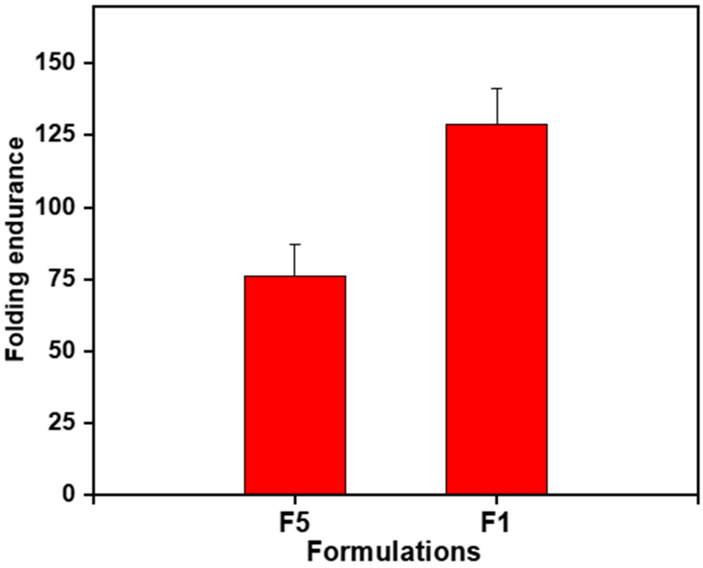

Figure S4. Effect of plasticizer concentration on folding endurance.

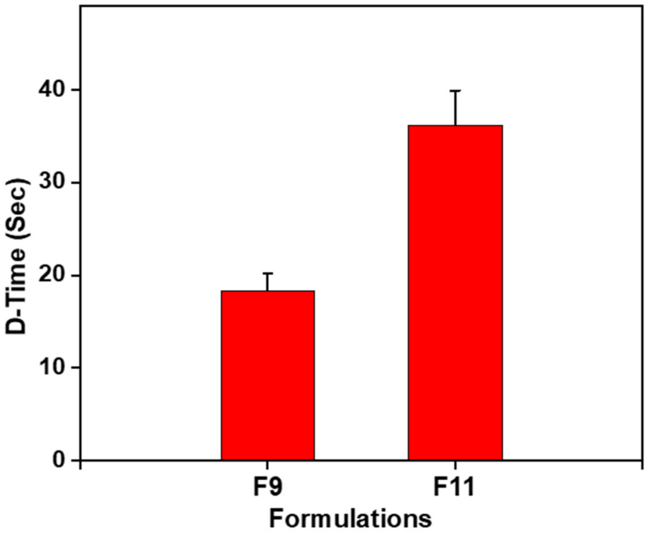

Figure S5. Effect of polymer concentrations on D-time.

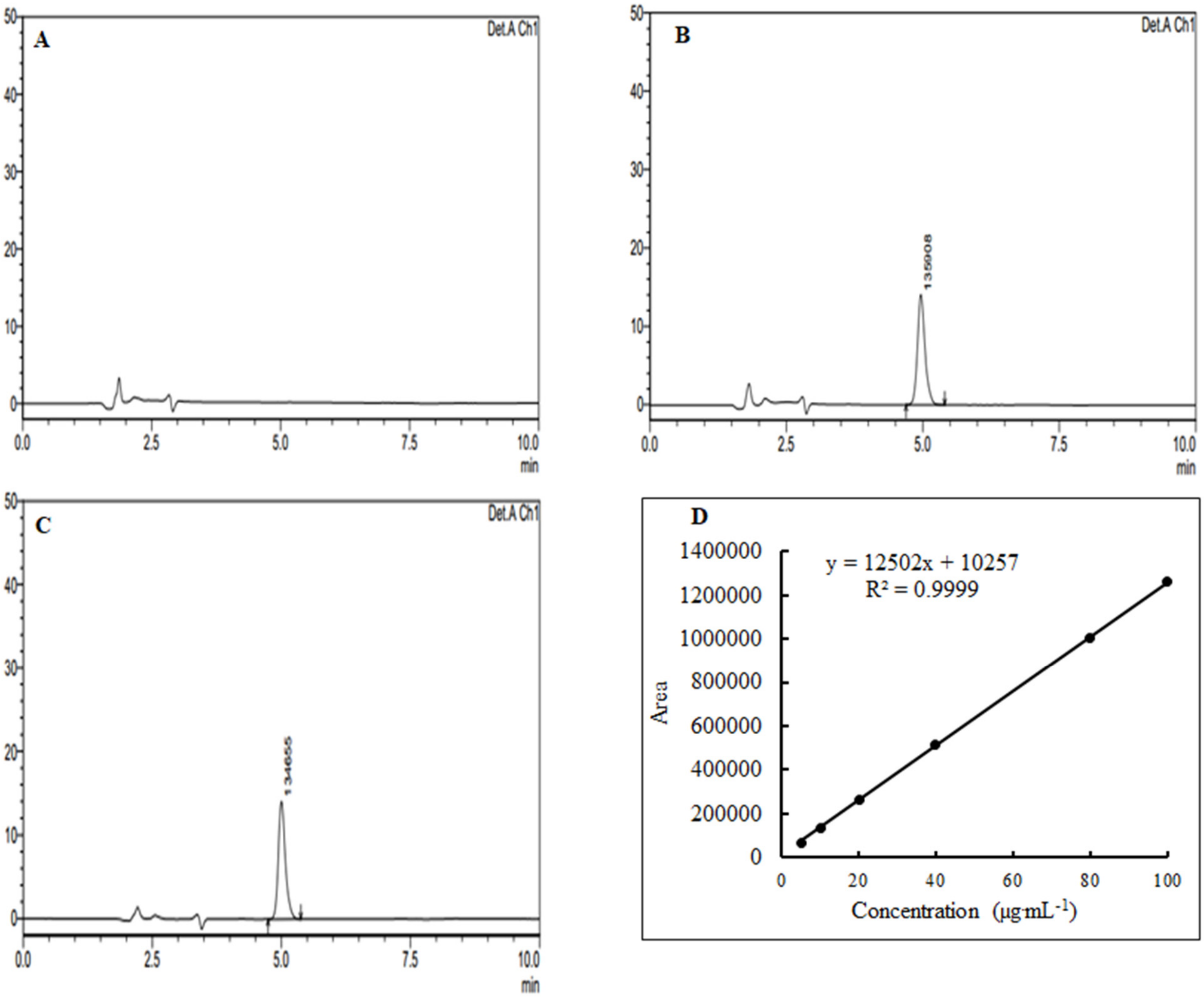

Figure S6. Typical chromatograms of blank solution (**A**), 10 μg·mL^{-1} standard solution (**B**), sample obtained after dissolution of ZMT-OFDFs (F1) at 5 min (**C**), typical calibration curve of ZMT in simulated saliva at pH 6.8 for in-vitro studies (**D**).

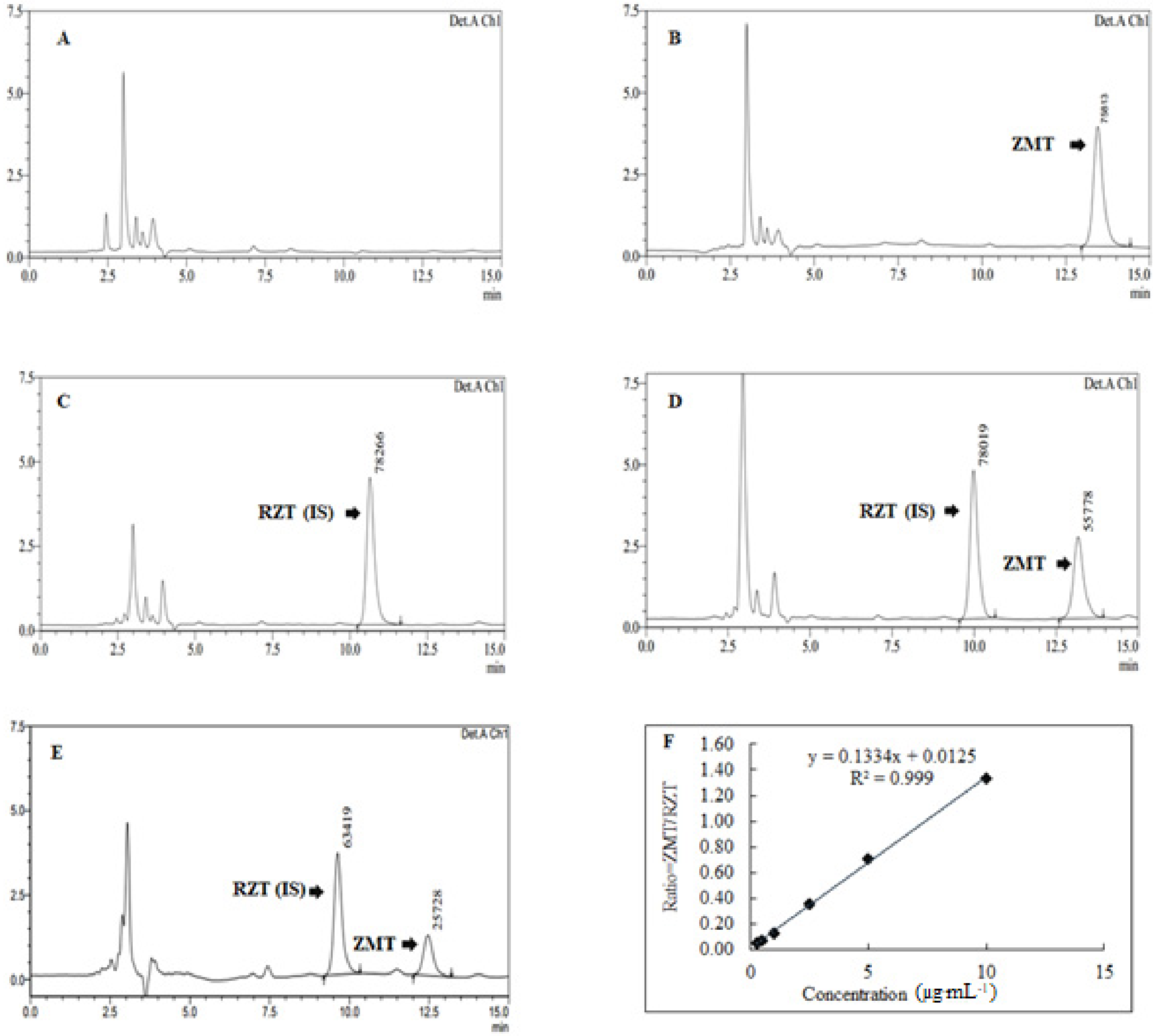

Figure S7. Representative chromatogram of blank rat plasma (**A**), plasma spiked with 10 μg·mL^{-1} ZMT (**B**), plasma spiked with 10 μg·mL^{-1} RZT (**C**), plasma spiked with ZMT-RZT (**D**), plasma sample taken at 0.5 h after oral administration of ZMT-OFDFs to rats (**E**), and a selected calibration curve of ZMT for in-vivo studies (**F**).

Table S1. Feasibility, pH, and drug content (%) determination of ZMT-OFDFs.

Film Code	Stickiness	Surface Appearance	Film Clarity	Drug Content (%)	pH
F1	Non-sticky	Uniform	Clear	96.4 ± 2.9	6.5 ± 0.5
F2	Non-sticky	Uniform	Clear	98.7 ± 2.8	6.1 ± 0.2
F3	Non-sticky	Uniform	Clear	100.1 ± 4.3	6.6 ± 0.7
F4	Non-sticky	Uniform	Clear	99.1 ± 2.6	6.8 ± 0.3
F5	Non-sticky	Uniform	Clear	98.2 ± 2.3	6.7 ± 0.8
F6	Non-sticky	Uniform	Clear	95.3 ± 2.3	6.8 ± 0.7
F7	Non-sticky	Uniform	Clear	99.6 ± 4.5	7.0 ± 0.2
F8	Non-sticky	Uniform	Clear	98.8 ± 4.2	6.9 ± 0.6
F9	Non-sticky	Uniform	Clear	102.9 ± 2.4	6.7 ± 0.7
F10	Non-sticky	Uniform	Clear	97.5 ± 2	6.3 ± 0.3
F11	Non-sticky	Uniform	Clear	99.9 ± 3.7	6.4 ± 0.5
F12	Non-sticky	Uniform	Clear	99.5 ± 5.5	6.2 ± 0.1
F13	Non-sticky	Uniform	Clear	96.6 ± 3.9	6.7 ± 0.8
